# Prévalence du trachome dans les zones de santé de Popokabaka et Kasongolunda dans la province du Kwango en République Démocratique du Congo, une étude quantitative

**DOI:** 10.11604/pamj.2025.50.45.44440

**Published:** 2025-02-09

**Authors:** Francisca Fataki Kimwesa, Prince Kimpanga Diangs, Jean Paul Tambwe, Felix Makangila, Jean Kalenga Nkashama

**Affiliations:** 1 Field Epidemiology and Laboratory Training Program, Kinshasa School of Public Health, Kinshasa, Democratic Republic of Congo,; 2 Department of Epidemiology and Biostatistics, Kinshasa School of Public Health, Kinshasa, Democratic Republic of Congo,; 3 Ministry of Public Health, Hygiene and Prevention, National Program for the Control of Neglected Tropical Diseases with Preventive Chemotherapy, Kinshasa, Democratic Republic of Congo

**Keywords:** Trachome, prévalence, trichiasis, follicules, Trachoma, prevalence, trichiasis, follicles

## Abstract

**Introduction:**

le trachome est un problème de santé publique majeure au monde. L´Afrique est le continent le plus touché et la République Démocratique du Congo (RDC) est confrontée aux complications oculaires dues à cette pathologie. Les données de la cartographie partielle du trachome en RDC attestent qu´il est généralisé, justifiant d´autres études. L´objectif de cette étude est de décrire le profil épidémiologique et clinique du trachome dans deux zones de santé du sud-ouest de la RDC.

**Méthodes:**

une enquête de prévalence du trachome a été menée en février 2023 dans les zones de santé de Popokabaka et Kasongolunda dans la province du Kwango, en RDC. Un échantillon de 20 villages a été sélectionné par sondage en grappe proportionnelle à la taille. Dans les ménages sélectionnés dans chaque village, toutes personnes âgées de 1 à 9 ans ainsi que celles de 15 ans et plus étaient examinés suivant le système de classement simplifié de l'OMS. Les données ont été soumises à des statistiques descriptives et le calcul des prévalences.

**Résultats:**

la prévalence du Trachome folliculaire chez les enfants de 1 à 9 ans était de 8,9% et celle du trichiasis trachomateux chez les sujets de 15 ans et plus de 0,1%.

**Conclusion:**

cette prévalence dépasse le seuil critique de l´OMS (5%). Les interventions appropriées aux communautés concernées sont nécessaires pour élimination le trachome en 2030.

## Abstract (en)

**Introduction:**

trachoma is a major public health problem worldwide. Africa is the continent most affected, and the Democratic Republic of Congo (DRC) is confronted with ocular complications due to this pathology. Data from the partial mapping of trachoma in the DRC show that it is widespread, justifying further studies. The aim of this study was to describe the epidemiological and clinical profile of trachoma in two health zones in southwest DRC.

**Methods:**

a trachoma prevalence survey was conducted in February 2023 in the Popokabaka and Kasongolunda health zones in Kwango province, DRC. A sample of 20 villages was selected by cluster sampling proportional to size. In the households selected in each village, all people aged 1 to 9 and those aged 15 and over were screened using the World Health Organization (WHO) simplified classification system, and the data were subjected to descriptive statistics and prevalence calculations.

**Results:**

the prevalence of follicular trachoma in children aged 1 to 9 was 8.9%, and that of trachomatous trichiasis in subjects aged 15 and over was 0.1%.

**Conclusion:**

this prevalence exceeds the WHO critical threshold (5%). Interventions appropriate to the communities concerned are needed to eliminate trachoma by 2030.

## Introduction

Le trachome constitue la première cause de cécité évitable au monde. L'Organisation Mondiale de la Santé (OMS) estime que 136 millions de personnes sont à risque de contracter le trachome dans le monde; 1,9 million de personnes sont aveugles ou malvoyantes; et 3,2 millions de personnes ont besoin d'une intervention chirurgicale, pour éviter la cécité trachomateuse au monde [1]. Le trachome est une infection bactérienne de l´œil; une kérato-conjonctivite d´évolution chronique caractérisée par la présence de follicules, une hyperplasie papillaire et un « pannus » cornéen évoluant jusqu´à la cécité provoquée par des infections répétées à Chlamydia trachomatis (OMS, 1962) [2]. Le trachome est un problème de santé publique dans 44 pays d´Afrique, d´Amérique centrale, d´Amérique du Sud, d´Asie, en Australie et du Moyen-Orient. Dans l´ensemble, l´Afrique reste le continent le plus touché et celui où les efforts de lutte sont les plus intensifs [2]. Selon les enquêtes de base réalisées en République Démocratique du Congo (RDC) dans 157 zones de santé endémiques pour le trachome entre 2014 à 2020, 81 ZS avaient des prévalences supérieures au seuil critique de l´OMS (supérieure à 5%). Ces enquêtes ont montré que 12 234 821 personnes sont à risque de contracter le trachome, 50046 cas étaient éligibles à la chirurgie pour éviter la cécité due au trachome en RDC [3,4]. Le trachome est considéré comme un problème de santé publique lorsque la prévalence de l'inflammation trachomateuse-folliculaire (TF) chez les enfants de 1 à 9 ans est égal ou supérieur à 5%, et lorsque le trichiasis trachomateux (TT) chez des personnes âgées de 15 ans et plus est égale ou supérieure à 0,2% [5,6].

La suppression du trachome en tant que problème de santé publique est une cible mondiale qui a été approuvée par l'Assemblée Mondiale de la Santé en 1998, et cela en utilisant la stratégie « CHANCE »: la chirurgie, les antibiotiques, la propreté du visage et l'amélioration de l'environnement [7-10]. Avant la mise en œuvre de la stratégie CHANCE, des enquêtes de base sur la prévalence du trachome sont recommandées [11]. En République démocratique du Congo, les données de la cartographie partielles du trachome attestent que le trachome est généralisé en RDC, justifiant ainsi d´autres études. Les ZS de Popokabaka et Kasongolunda avec des prévalences respectivement de 17,4% et 19, (4,9%) en Trachome folliculaire (TF) à l´appréciation rapide (4) sont éligible à la cartographie de base. C´est dans ce cadre que la présente étude s´était proposée de déterminer la prévalence du Trachome folliculaire et trichiasis trachomateux dans les zones de santé de Popokabaka et Kasongolunda pour guider le ministère de la santé à fournir les interventions appropriées aux communautés concernées.

## Méthodes

**Conception de l´étude:** il s´est agi d´une étude transversale descriptive de population organisée entre le 20 février au 13 mars 2023.

**Cadre de l´étude:** la présente enquête de ménage a été organisée entre le 20 février au 13 mars 2023 dans deux zones de santé du sud-ouest du pays (Popokabaka et Kasongolunda). En effet, la RDC est administrativement subdivisée en 26 provinces dont chacune a une division provinciale de la santé (DPS) qui comprend à son tour plusieurs zones de santé (ZS). L´enquête de ménage a été conduite dans les ZS de Popokabaka et Kasongolunda dans la DPS du Kwango, une province du sud-ouest de la RDC.

**Participants à l´étude:** la population d´étude était constituée des personnes âgées de 1 à 9 ans ainsi que celles de 15 ans et plus, résidant dans les ménages des villages sélectionnés dans les 2 ZS ciblées. Toute personne de ces tranches d´âges, ayant consentie à l´enquête était incluse dans l´étude. Les ménages où il y avait des absents, étaient revisités le même jour. Les personnes absentes pendant la deuxième visite étaient exclues de l´étude.

**Variables:** les variables d´intérêt pour la présente étude étaient la taille de la population totale du village, le sexe, l´âge, la présence du trachome folliculaire chez les enfants de 1 à 9 ans, la présence du trichiasis trachomateux chez les sujets de 15 ans et plus.

**Variable quantitative:** l´âge de participant.

**Sources des données/mesure:** les données de la présente étude ont été collectées par l´interview, l´observation et l´examen du trachome. Un questionnaire électronique de l´application Tropical Data a été utilisé au moyen des téléphones Android. Cette enquête était réalisée par 10 enquêteurs regroupés en 5 binômes formés chacun d´un examinateur, technicien supérieur en ophtalmologie (TSO) et d´un opérateur de saisi. L´équipe d´enquêteurs disposait de la liste de 20 villages sélectionnés pour l´étude. Une fois dans le village où les 30 ménages étaient sélectionnés, le binôme d´enquêteurs procédait par les civilités au chef du village et son équipe. Dans chaque ménage sélectionné, le chef du ménage donnait son consentement à participer à l'enquête. Les enquêteurs arrivaient tôt au ménage pour ne pas rater les participants. Ensuite, tous les membres du ménage éligible à l´enquête passaient à l'examen du trachome après consentement. L´examen était effectué en utilisant le système de classement simplifié de l'OMS. La paupière, la conjonctive tarsienne et la cornée étaient examinées à l'aide d'une loupe grossissante x2.5, d'une lampe torche, des instruments de mesure des follicules pour rechercher les signes de trachome folliculaire (TF), l´inflammation trachomateuse-intense (TI) et ses complications: trichiasis trachomateux (TT) et cicatrices trachomateuse (TS) pour tous les cas de trichiasis observés. Pour les cas de trichiasis, des questions sur son traitement étaient posées.

**Figure 1:**
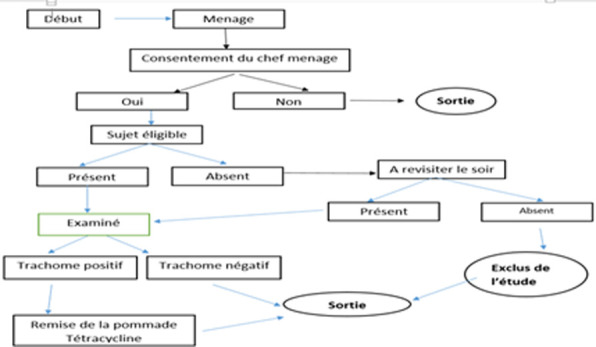
représentation schématique de la collecte des données dans le ménage

Pour confirmer la réponse aux questions sur la gestion du trichiasis, l'examinateur cherchait des preuves d'une cicatrice chirurgicale. Il observait le trichiasis chez le participant. Il vérifiait la présence d'une cicatrice chirurgicale de trichiasis tout en retournant la paupière pour chercher le TF, TI and TS. Les opérateurs de saisi remplissaient les questionnaires dans les Smartphones. A la fin de l´enquête dans chaque ménage, les enquêteurs donnaient 2 tubes de pommade Tétracyclines pour les yeux, aux parents ou tuteurs des enfants trouvés atteints de TF ou TI et toutes les personnes atteintes de trichiasis étaient référées au centre de santé approprié pour une chirurgie. La codification simplifiée de l´OMS se résume comme suit, trachome inflammatoire folliculaire (TF): consiste en la présence d´au moins 5 follicules sur la conjonctive tarsienne supérieure, trachome inflammatoire Intense (TI): est caractérisée par l´épaississement inflammatoire prononcée de la conjonctive tarsienne qui masque plus de la moitié des vaisseaux profonds du tarse. Trachome cicatriciel (TS): se traduit par la présence d´un tissu de cicatrisation nettement visible sur la conjonctive tarsienne, trichiasis trachomateux (TT): est défini par un ou plusieurs cils frottant le globe oculaire. Les cils épilés sont aussi une évidence d´un trichiasis. La présence d´opacité cornéenne (CO): opacité cornéenne évidente recouvrant l´aire pupillaire [12,13]. En une journée, un binôme terminait l'enquête dans une grappe. Cinq jours de travail étaient nécessaires pour l´unité d´évaluation (UE) Figure 1.

**Bias:** le biais de sélection était assuré par la représentativité de l´échantillon (taille suffisante et échantillonnage probabiliste), les critères d´inclusion et la réduction le plus possible des non réponses et des refus, par une explication claire aux participants sur l´étude et leur intérêt à l´étude. Pour le biais d´information, une aide-mémoire a été rédigé et partagée aux enquêteurs. Ces derniers ont été formés et supervisés, les instruments de collecte des données (questionnaires) étaient pré-testés.

**Taille de l’échantillon:** la taille de l'échantillon a été calculée en supposant une prévalence attendue de 4% avec une précision absolue de ± 2%, un niveau de confiance de 95%, un effet du plan de sondage (DEFF) de 3, suivant les recommandations de l´OMS en utilisant formule de Schwartz [14]. N représente la taille minimale de l´échantillon; Z est le coefficient de confiance; p est la proportion supposée de la population cible ayant la caractéristique étudiée et d est le degré de précision absolu voulue.


$$N=\frac{z^{2}p\left(1-p\right)}{d^{2}}$$

Le nombre minimal de 971 enfants âgés de 1 à 9 ans était nécessaire pour l´étude. En tenant compte des non-répondants (10%), ce nombre a été majoré à 1068 enfants âgés de 1 à 9 ans. Pour atteindre ce nombre d´enfants dans les 2 zones de santé, le nombre de ménages et villages à visiter a été obtenu en considérant qu´en RDC, le nombre d´enfants par ménage est de 2,0 en moyenne. Et qu´un binôme d´enquêteurs peut examiner 30 ménages par jour (environ 60 enfants par jour). Vingt villages étaient donc nécessaires pour atteindre cet échantillon (60 multiplié par 20). Le sondage en grappe a été utilisé suivant les recommandations de l´OMS pour les enquêtes de prévalence du trachome [14-17].

Ce sondage a été réalisé à deux niveaux: au premier niveau, la sélection des grappes s´est faite en utilisant la base de sondage (liste des villages) fournie par la base des données du Programme National de Lutte contre les Maladies Tropicales Négligées à Chimiothérapie Préventive (PNLMTN - CTP). Vingt villages ont été sélectionnés en utilisant la technique de la probabilité proportionnelle à la population (PPP). Au deuxième niveau, la sélection des ménages et des participants dans chaque village s´est faite selon la méthode d'échantillonnage par segmentation. Chaque grappe sélectionnée était divisée en segments d'environ 30 ménages. Un seul segment était sélectionné par tirage au sort. Dans le segment sélectionné, tous les ménages étaient visités et tous les membres du ménage éligibles étaient examinés pour le trachome. Pour les villages ayant moins de 30 ménages, les ménages ont été complétés dans le village contigu.

**Méthodes statistiques:** les données collectées avec les smartphones, étaient transférées vers le serveur puis exportées sur Excel 2016. Après nettoyage, elles étaient exportées sur SPSS 27 pour analyses. Les statistiques descriptives (moyennes et écart-types) ont été utilisées pour résumer les variables quantitatives. Les prévalences de trachomes ont été calculées chez les enfants de 1 à 9 ans (TF) et chez les sujets de 15 ans et plus (TT).

**Considération éthique:** le protocole de cette étude a été soumis pour approbation au Comité d´éthique. Le consentement verbal pour participer à l'enquête était demandé aux chefs de ménage sélectionnés. Un consentement éclairé verbal en vue de l'examen était demandé à chaque participant à l'enquête ou à son parent ou tuteur pour les enfants. La confidentialité de résultat des examens des yeux était observée. Pendant les enquêtes, les noms des participants étaient enregistrés pour faciliter le processus d'examen (c'est-à-dire pour permettre aux participants d'être appelés par leur nom et de réduire la probabilité d'erreurs sur le terrain). Seule, l´équipe de recherche avait accès aux données. Les enfants trouvés avec un TF étaient traités avec la pommade ophtalmique (tétracycline). Toute personne atteinte de trichiasis était référée au centre de santé le plus proche. La sélection des participants étaient faites dans l´équité. Toutes les classes sociales étaient concernées.

## Résultats

**Caractéristiques des participants:** au total, 1610 enfants âgés de 1 à 9 ans sur les 1068 prévus et 1384 adultes de 15 ans et plus étaient enquêtés. Les sujets absents après la deuxième visite pour la plupart dans les travaux des champs n´étaient pas visités.

**Données descriptives:** parmi les enfants, l´âge moyen était de 5 ans avec un écart type de 2,5. Chez les adultes, l´âge moyen était de 36 ans avec un écart type de 15.

**Principaux résultats**

**Caractéristiques socio démographiques de l´échantillon:** l´enquêté de ménage a été réalisée dans 2 zones de santé regroupées en une unité d´évaluation. Au total 600 ménages dans 30 villages étaient enquêtés. Selon les tranches d´âge et le sexe, parmi les 1610 enfants de 1 à 9 ans, les garçons étaient légèrement plus représentés (51%) et la tranche d´âge de 5 à 9 ans représentaient plus de la moitié de l´échantillon (55,3%) (Tableau 1). Parmi les 1384 adultes, plus de la moitié de l´échantillon étaient représenté par les femmes (56%) et la tranche d´âge de 20 à 59 ans majoritaire (Tableau 2).

**Tableau 1:** répartition des enfants enquêtés selon les tranches d´âge et le sexe

Caractéristiques		Ensemble (n=1610)	Pourcentage
Sexe	Masculin	821	51
	Féminin	789	49
Tranche d´âge en années	1 à 5	890	55
	6 à 9	720	45

Parmis les enfants de 1 à 9 ans enquetés les garçons avaient legerement dépassé les filles et la tranche d´âge de 1 à 5 ans étaient majoritaire

**Tableau 2:** répartition des adultes enquêtés selon les tranches d´âge et le sexe

Caractéristiques		Ensemble (n=1384)	Pourcentage (%)
Sexe	Masculin	609	44
	Féminin	775	56
Tranche d´âge en années	15-20	217	16
	21-59	1048	75
	≥60	119	9

Dans l'échantillon des adultes, les femmes étaient majoritaires et le 3/4 était constitué de la tranche d´âge de 21 à 59

**Caractéristiques cliniques des sujets:** la prévalence de Trachome folliculaire chez les enfants de 1 à 9 ans était de 8,9%, les filles étaient plus affectées et les jeunes enfants 1 à 5 ans. Dans Tableau 3 la prévalence du trichiasis trachomateux chez les sujets de 15 ans et plus était de 0,1%; seules les femmes étaient affectées et dans les tranches d´âge de 20 à 59 ans et plus de 60 ans (Tableau 4).

**Tableau 3:** prévalence du trachome folliculaire chez les enfants de 1 à 9 ans et selon sexe

Caractéristiques		Ensemble	Trachome folliculaire
		(n=1610)	n (%)
Sexe	Masculin	821	72 (8,7)
	Féminin	789	72 (9,1)
Tranche d´âge en années	1 à 5	890	100 (11,2)
	6 à 9	720	44 (6,1)


Pour le trachome folliculaire, les filles et la tranche d´âge de 1 à 5 ans étaient plus atteint

**Tableau 4:** prévalence du trichiasis trachomateux chez les sujets de 15 ans et plus selon le sexe

Caractéristiques		Ensemble	Trichiasis Trachomateux
		(n=1384)	n (%)
Sexe	Masculin	609	0 (0,0)
	Féminin	775	2 (0,1)
Tranche d´âge en années	15 - 19	217	0 (0,0)
	20 - 59	1048	1 (0,09)
	≥60	119	1 (0,84)

Pour le trichiasis trachomateux, seules les femmes de 20 ans et plus étaient atteint

## Discussion

La prévalence du Trachome folliculaire chez les enfants de 1 à 9 ans (8,9%) était élevée et au-dessus du seuil critique de l´OMS (5%). Le trachome folliculaire est un problème de santé publique dans ces zones de santé. La prévalence du trichiasis trachomateux chez les adultes (0,1%) est inférieure au seuil critique de l´OMS (0,2%). Le trichiasis trachomateux n´est pas un problème de santé publique dans ces zones. Au-delà de plusieurs forces tels que les enquêteurs qualifiés, bonne formation par des superviseurs routiniers dans le domaine, prétexte du questionnaire, bonne supervision de la collecte, appui des prestataires du ministère et de l´école de santé publique dans l´activité quelques faiblesses ont été relevé tel que la communication sur terrain difficile suite à l´absence ou mauvais réseau téléphonique, enquête menée pendant la période pluvieuse rendant difficile la collecte. Un certain nombre des enquêtes de prévalence du trachome ont été mené en RDC dans d´autres zones de santé, organisé par le ministère de la santé publique mais sans appui de l´école de santé avec la même méthodologie cad utilisant le système de codification de l´OMS pour l´examen des yeux.

La prévalence dans notre étude reste inférieure à celles observées dans les enquêtes précédentes menées dans certaines zones de santé comme Pweto (41,6%), Lita (19,4%), Minova (15,2%). Elle est cependant supérieure à celles des autres zones de santé de la RDC: Jiba (7,5%), Drodro (6%), Lomela (4,8%). De même pour le TT ou des prévalences en TT avaient varié entre 5,09% (Angumu) et 0% (Djombo, Lomela, Mitwaba...). Ces différences entre les résultats de la présente enquête et celles des autres zones de santé peuvent être réelles, les approches méthodologiques étant les mêmes. Dans certaines zones de santé du pays tel que Minova, Libenge, Kilwa la prévalence en TT suit celle de TF. Ceci est normal car la TT reste la conséquence de TF. Mais dans notre cas c´est le contraire, Il est important de chercher à expliquer la discordance entre la prévalence élevée du trachome actif (TF) qui fait du Trachome un problème de santé publique dans cette région et la faible proportion des cas de complication (TT). Tout en sachant que dans les 2 zones de cette étude, aucune intervention contre cette maladie est mise en œuvre jusqu´à présent.

Les différences de prévalences observées dans les zones de santé en RDC sont similaires à la situation du Mali entre les régions pour le Trachome folliculaire: Kayes (42,5%), Ségou (23,1%) [18-22]. Au vu de nos résultats, le trachome folliculaire est un problème de santé publique dans les deux zones de santé enquêtés. Ceci confirme que la maladie est généralisée en RDC et des études sont nécessaire pour les zones non encore enquêtées. Il est important de mener une étude sur les facteurs qui influent sur ces prévalences de trachome folliculaire pour la mise en œuvre des interventions ainsi que sur les facteurs qui expliquerait cette discordance entre prévalence de trichiasis trachomateux et celle du trachome folliculaire.

**Limite de l´étude:** bien que l´organisation de ce travail a permis une bonne participation des sujets à l´activité, nous avons noté quelques limites notamment: certains sujets absents pour les travaux des champs n´ont pas participé à l´étude malgré la deuxième visite; sans négliger l´instabilité ou absence des réseaux téléphoniques dans les villages rendant difficile la communication et l´envoi journalier des formulaires au serveur bien que ce dernier n´a pas eu d´impact sur le résultat; et le mauvais état des routes et les pluies qui ont allongé le nombre des jours sur terrain. Néanmoins la taille prévue de l´échantillon était atteint et le résultat peut être généralisé dans ces zones.

## Conclusion

La prévalence du trachome (FT) observée dans cette étude est élevée et permet de confirmer que le trachome est un problème majeur de santé publique dans les zones de santé de Popokabaka et Kasongolunda. Pour atteindre la cible mondiale de son élimination d´ici 2030, le ministère de la santé devrait mettre en place la stratégie CHANCE. Des enquêtes supplémentaires de prévalence sont nécessaires pour évaluer l´importance de cette pathologie dans d´autres zones de santé de la RDC.

### Etat des connaissances sur le sujet

- Le trachome constitue un problème de santé publique dans 42 pays et est responsable de cécité ou de déficiences visuelles pour environ 1,9 million de personnes; la cécité due au trachome est irréversible. Selon des données de juin 2022, 125 millions de personnes vivent dans des zones où le trachome est endémique et risquent la cécité (OMS 2022);
- En République Démocratique du Congo, selon les enquêtes de base réalisées dans 157 zones de santé pour le trachome entre 2014 à 2020, 81 ZS avaient des prévalences supérieures au seuil critique de l’OMS (supérieure à 5%);
- Les zones de santé de Popokabaka et Kasongolunda avaient des prévalences respectivement de 17,4% et 19,9% en Trachome folliculaire (TF) à l´appréciation rapide.

### Contribution de notre étude à la connaissance

- L´étude apporte la connaissance du profil épidémiologique du Trachome dans les zones de santé de Popokabaka et Kasongolunda pour guider le ministère de la santé à fournir les interventions appropriées aux communautés concernées.

## Remerciements

A l´école de santé publique de Kinshasa car c’est dans le cadre de la formation en épidémiologie de terrain et laboratoire (FLTP) qu´elle a été réalisée. Au professeur Prince Kimpanga Diangs, le directeur de ce travail qui malgré ses multiples occupations a apporté son appui et les connaissances nécessaires à l´élaboration de ce travail. Aux collègues du programme National de lutte contre les maladies tropicales négligées pour leur accompagnement pendant la collecte des données et nettoyage des données.

## References

[1] World Health Organisation (2020). World Health Organisation Alliance for the Global Elimination of Trachoma by 2020. WHO.

[2] Jean-François Schémann (2013). Le trachome une maladie de la pauvreté. IRD éditions.

[3] Ministere de la Sante (2016). Plan stratégique de lutte contre les maladies tropicales négligées à chimiotherapie préventive 2016-2020 négligées Burkina Faso.

[4] Kilangalanga J, Ndjemba JM, Uvon PA, Kibangala FM, Mwandulo JSB, Mavula N (2018). Trachoma in the Democratic Republic of the Congo: Results of 46 Baseline Prevalence Surveys Conducted with the Global Trachoma Mapping Project. Ophthalmic Epidemiol.

[5] Organisation mondiale de la Santé (2018). Paramètres de conception des enquetes de prévalence du trachome en population. OMS.

[6] Organisation mondiale de la Santé (2012). Rapport De La Seizième Réunion De l´ Alliance Oms Pour L’élimination Du Trachome Cécitantd´ Ici 2020. OMS.

[7] World Health Organization (2016). Validation of Elimination of Trachoma as a Public Health Problem. Geneva.

[8] Organisation mondiale de la santé (2012). Rapport De La Seizième Réunion De L´Alliance OMS pour l’élimination du Trachome cécitant d’Ici. OMS.

[9] Thylefors B, Négrel AD, Pararajasegaram R (1992). La surveillance épidemiologique du trachome, bilan et perspectives. Rev Int Trach Pathol Ocul Trop Subtrop Sante Publique.

[10] Paul E, Laura F, Robin B, David M (2006). Mise en Oeuvre de la Stratégie CHANCE dans la lutte contre le Trachome: Boîte à outils avec des interventions pour la promotion du nettoyage du visage et changement de l´environnement. ITI.

[11] Organisation mondiale de la santé (1996). Planification pour l’élimination mondiale du trachome à l´échelle mondiale. Genève.

[12] Scheman JF, Sacko D, Malvy D, Momo G, Traore L (2002). Risk factor for trachoma in Mali. Int J Epidemiol.

[13] Modibo Dao (2013). Trachome dans le cercle de Banamba après 6 ans de mise en oeuvre de la stratégie CHANCE: Résultat de l'enquête 2012. These de Médecine.

[14] Telly M (2020). Etude de la prevalence du trachome dans le district sanitaire de Markala en 2019. (Doctoral dissertation USTTB).

[15] Solomon AW, World Health Organization, International Trachoma Initiative (2006). Lutte contre le trachome: un guide pour les gestionnaires de programme. Organisation mondiale de la santé.

[16] Thylefors B, Dawson CR, Jones BR, West SK, Taylor RH (1987). A simple system for the assessment of trachoma and its complications. Bull World Health Organ.

[17] Oumar S (2009). Le trachome dans la region de koulikoro apres 4 ans de pause therapeutique: resultats de l´enquete de 2009. ADHL.

[18] Schémann JF, Sacko D, Banou A, Bamani S, Boré B, Coulibaly S (1998). Cartographie du trachome au Mali: resultats d'une enquete Nationale. Bull World Health Organ.

[19] Keita F (2011). Etude de l´impact de la mise en oeuvre de la stratégie CHANCE dans le district sanitaire de Barouéli au Mali: Résultat d´enquête 2010. ADHL.

[20] Bakary Touré (2014). Trachome dans le district sanitaire de Bafoulabe au Mali: resultats de l´enquete sousdistrict 2013. ADHL.

[21] Amadou Dit Karamoko (2012). Surveillance post endemique du trachome dans le district sanitaire de niono: resultat d´enquete 2010. ADHL.

[22] Youssouf Traoré (2005). Enquete epidemiologique sur le trachome dans le cercle de San en 2005. ADHL.

